# Mellin’s Food

**Published:** 1881-08-20

**Authors:** 


*Mellin's Food for Infants and Invalids.*—We invite special attention to the advertisement of Mellin's Food for Infants to be found in this journal. We hope our medical friends will test the article. There surely is abundant need for such an article of food as this is described to be.

